# Translating research into practice: Lessons from trials of thrombolysis in acute stroke

**Published:** 2008

**Authors:** Graham Venables

**Affiliations:** Sheffield Teaching Hospitals NHS Foundation Trust, Honorary Professor of Vascular Neurology, University of Sheffield, President, Association of British Neurologists

**Keywords:** Ischemic stroke, thrombolytic therapy

## Abstract

There exist individual, institutional and national barriers to change, none more so than when introducing new therapies into medical practice especially those that involve organizational change. This paper, presented as an address to the joint meeting of the Association of British Neurologists and the Indian Academy of Neurology in October 2007, explores these barriers in the context of the instruction of thrombolytic therapy for patients with acute ischaemic stroke and suggests how they might be overcome using evidence from relevant clinical trials and observational studies.

The notion that revascularizing the ischemic brain might improve outcome in stroke patients is in itself deceptively simple to the point of being naïve; yet this idea, along with the equally simple notion that sleeping neurons, as opposed to dead neurons, might be woken up by restoration of oxygen and glucose, remains the bedrock of therapy in acute ischemic stroke. Without these measures it is unlikely that other protective modalities will prove effective.

That there is a significant burden from acute stroke is another given, with stroke being the third most common cause of death worldwide and the most common cause of persisting disability. In the UK incidence rates for first ever stroke are between 119 – 203/100,000; in India rates are probably comparable in urban communities but possibly double in rural areas with hypertension being the most common risk factor leading to more hemorrhagic stroke than would be expected in the West. Given the size of the population, this translates into a vastly greater prevalence of stroke-related disability in India than in the United Kingdom.

It is against this background that attempts have to be made to reduce the burden of stroke. Without doubt, in both countries primary prevention has to take first place. However, for those treating atheroembolic stroke, there is also the challenge of translating science into action.

Early attempts to introduce thrombolysis were doomed to failure because of the lack of accurate imaging techniques. Meyer *et al*. reported disappointing results in 1963 and 1964[1] at the same time that Shaw *et al*.[2] stopped their trial of anticoagulants in stroke because of increased rates of bleeding. In 1964, a whole edition of the British Medical Bulletin was given over to the possible uses of antifibrinolytic therapy, with no mention of its possible use in ischemic stroke or of Meyer's recent publications.

The successful introduction of new technologies requires an understanding of the barriers that exist and the availability of strategies for managing these barriers, be it at individual, local, regional, or national level.

Evidence of effectiveness is of course important, but often there are other requirements that have to be met. Parallels exist with the introduction of thrombolytic therapy for myocardial infarction, where many tens of thousands were randomized in clinical trials before there was widespread acceptance of the therapy.

At the level of the individual, the barriers to the acceptance of a novel therapy include lack of awareness of areas of changing practice; but this probably does not apply in stroke medicine in which, in the UK at least, there has been the widely heralded and welcome launch of the National Strategy for Stroke though with some confusion resulting from conflicting guidelines and protocols. Some individuals regard such guidelines as medicine by cookery-too rigid, biased, and impractical, and challenging their autonomy as clinicians. Others lack confidence in the guideline developer, disagree with the evidence, or disregard them as being not applicable to their patients. Other barriers to change include patient preferences, lack of time or resources, poor motivation, fear of litigation, and organizational constraints.

In the field of thrombolysis there is some favorable evidence, but more is required. In North America, the NIH Study[3] (reported in 1995) recruited 624 patients presenting within 3 h of onset of their stroke (as judged on clinical grounds) after CT scanning had excluded a bleed or stroke mimic. The results suggested that there were 120–160 more independent survivors per 1,000 treated patients and that the risk of intracranial hemorrhage was minimal. The study has been reanalyzed many times and has demonstrated benefit across all severities of stroke. The second European Cooperative Acute Stroke Study (ECASS II)[4] recruited 800 patients aged 18–80 years within 3 h of onset of stroke. CT was used to exclude brain hemorrhage, stroke mimic, or patients with ischemia involving greater than one third of middle cerebral artery (MCA) territory. There was a 3.7% absolute difference in favor of treatment with recombinant tissue plasminogen activator (rTPA) and 37 more independent survivors per 1,000 treated patients. Two large trials are ongoing. ECASS III is in the process of recruiting 800 patients aged 18–80 years 3–4.5 h after onset of stroke; this study has a 90% power to detect a 10% difference between treatment groups. The Third International Stroke Trial (IST3) aims to recruit 3000 patients worldwide and will report results after 2011. An updated Cochrane review of all randomized trials[5] suggested that there was a 20% (95% CI: 7 to 23%) reduction in death/dependency; however, there was significant heterogeneity (I2–62%), suggesting that robust conclusions about the use of thrombolytic drugs cannot yet be drawn.

One of the central issues when developing hyperacute services for people with stroke is related to the period after stroke onset when any treatment might be effective. It is widely believed that treatment with rTPA does not work after 6h after onset of stroke. What is clear from the data available on treatment of individual patients up to 3 h after stroke onset is that any assumptions about effectiveness of treatment after 3 h require validation though the acquisition of additional evidence, from randomized clinical trials, including ECASS III (since submission of this paper the results of ECASS III have indicated some benefit in treating selected patients up to 4.5h after onset of stroke) and IST3.

What is also clear is that even after completion of the clinical trials and the subsequent implementation of thrombolysis, many patients remain without access to services. In North America, no eligible patients were treated in 35% hospitals with rTPA; the use of thrombolysis was particularly low if a neurologist was not available. Female patients and African-Americans were less likely to be treated, as also the elderly, i.e., those who are most likely to benefit.[6] By extrapolation this would suggest that nearly 50,000 patients in the UK are denied access to treatment under the terms of the license for administration of tPA; IST3 includes older patients and has already increased the evidence on the benefit of thrombolysis in older people by eight fold.

In summary therefore, evidence from the trials conducted to date tell us that treatment is associated with 7% more fatal and nonfatal brain hemorrhages, 6% more early deaths but, also, 12% fewer deaths or dependent people overall. There is however significant heterogeneity between trials suggesting that caution should be exercised in interpreting the results. If the results are reliable, it means that there should be one additional patient alive and independent for every 7–10 treated patients.

We still do not know what to do for people with stroke in whom IV rTPA may be of benefit but when there is no evidence to support its use, for example, those who present within 3 h of stroke onset but do not exactly meet the trial entry criteria, e.g., patients aged > 80 years, all patients who present after 3 h and within 6 h of stoke onset, patients with severe stroke or mild strokes, and those with subtle early ischemic change or leucoaraiosis on CT. In addition there are uncertainties about the delivery of hyperacute therapies within other health care systems, such as the NHS in the UK or those in rural India.

Even where evidence is missing there are ways of obtaining consensus, e.g., the Delphi technique. A recent review from the Netherlands[7] was unable to achieve consensus over the effectiveness of treatment for patients who present between 3 – 4.5h after stroke onset, patient age, and timing of recent major surgery or recent arterial puncture. Patients who were improving and blood pressure reduction. It did achieve consensus regarding excluding from treatment those with stroke within the last 1.5 months, head trauma within 2 months, gastrointestinal surgery and urinary tract hemorrhage within the last 14 days, stroke severity < NIH 3, systolic blood pressure >185 mmHg and diastolic blood pressure >110 mmHg, a platelet count < 90/10^6^, glucose > 2.6 mmol/l and random glucose < 22 mmol/l, and international normalised ratio > 1.5 or activated partial thromboplastin time < 50.

Institutional barriers to change are rife within health care systems and related to institutional policies, the effects of changing or displacing existing practice, financial pressures, and the climate of clinical and research governance. In the UK, policy was driven by local champions until the year 2000 when stroke was introduced into the National Service Framework for older people. Implementation was haphazard and ongoing concerns about the maturity of stroke services has resulted in the development of the National Strategy for Stroke[8] that was launched in March 2008.

The costs associated with changes in practice can be significant but in England the introduction of thrombolysis on license has been further facilitated by an additional top-up tariff under Payment by Results, the mechanism by which NHS trusts are reimbursed for clinical activity. The costs of further research have been supported to some extent by the development of government-funded stroke research networks but new trials are heavily handicapped by the requirements of the European Research Directive, the activities of the Medicines and Healthcare products Regulatory Agency (MHRA), local research ethics committees, radiation protection agencies, the 2008 Mental Capacity Act, and sponsorship and legal agreements between organizations sponsoring and participating in research activities. The effect of these was demonstrated in a recent review of the time taken to approve research governance applications in 50 UK NHS hospital trusts; which showed that there was up to 2 years’ delay in initiating research projects.[9]

SITS-MOST (Safe Implementation of Thrombolysis in Stroke Monitoring Study) demonstrated substantial variation in the use of thrombolytic therapies across Europe. Finland has the highest uptake, with up to 30% being treated. Among the European Union countries, the United Kingdom falls in the lowest quartile. National barriers to change include those relating to resource allocation, licensing, education, and measurement of outcome. The National Institute for Health and Clinical Excellence has addressed some of these barriers in the UK and has recommended that alteplase with best supportive care is cost-effective when compared with placebo with best supportive care for people with acute stroke.[10] For the granting of a license to use rTPA in acute stroke the European Union regulatory authorities required that a monitoring cohort be set up (in the view of many a wasted opportunity for a large clinical trial). The aim of SITS-MOST was to assess the safety and efficacy of intravenous alteplase as thrombolytic therapy when given within the first 3 h of onset of acute ischemic stroke. Between 2002 and 2006, 6483 patients were recruited from 285 centers in 14 countries for this prospective, open, monitored, observational study. Primary outcomes were symptomatic intracranial haemorrhage (ICH), deterioration in NIH stroke scale score by ≥ 4 within 24 h, and mortality at 3 months. Mortality rate, the proportion of patients with symptomatic intracranial haemorrhage (SICH), and functional outcome at 3 months were compared with relevant pooled results from randomized controlled trials.[11] The results confirmed the findings of the randomized trials and a license was granted in 2005.

In India, the use of thrombolytic drugs has been approved by regulatory authority and pilot studies have started, including the launch of IST3 at the All India Institute of Medical Sciences (AIIMS). Inevitably however, there are significant problems in getting patients to treating institutions on time, including poor awareness regarding stroke in the general population and the cost of the drug/treatment.

In summary, a national strategy for implementation requires national guidelines and a national will to effect change. A cost–benefit analysis needs to be done. Local opinion leaders need clinical decision–making tools and help with monitoring compliance and outcomes though audit. In the UK this has been possible through the UK Stroke Strategy, which highlights the need for early identification of suitable patients by paramedical staff and their urgent transfer to expert treatment centers that are open 24 h a day. Such centers must be able to offer immediate imaging, same-day admission to a stroke unit, and access to specialist care for complex cases. Local opinion leaders receive assistance through the work of the British Association of Stroke Physicians,[12] involvement in clinical research networks and trials such as IST3,[13] and the guideline development work of the National Institute for Health and Clinical Effectiveness and the Royal College of Physicians,[14] and the Sentinel Stroke Audit.

What, then, are the key messages to setting up a stroke service?

- Walk before you run; set up a good stroke rehabilitation unit for which there is overwhelming evidence of effectiveness, ensure you have fast access to good CT imaging, and then give people with cerebral infarction some aspirin to reduce the incidence of early stroke recurrence.
- Build capacity in acute stroke care; train more stroke physicians, and encourage neurologists to work with elderly care physicians.
- Participate in trials of IV thrombolysis that provide a governance umbrella and help train and monitor new centers.
